# Effect of Allogeneic Oral Mucosa Mesenchymal Stromal Cells on Equine Wound Repair

**DOI:** 10.1155/2021/5024905

**Published:** 2021-12-14

**Authors:** Paola Di Francesco, Pauline Cajon, Christophe Desterke, Marie-France Perron Lepage, Jean-Jacques Lataillade, Tewfik Kadri, Olivier M. Lepage

**Affiliations:** ^1^Unité ICE-Groupe de Recherche en Médecine et Rééducation des Equidés de Sport (GREMERES), Centre for Equine Health, Ecole Nationale Vétérinaire de Lyon, VetAgro Sup, Université de Lyon, Marcy l'Etoile 69280, France; ^2^Stem Cell Vet Therapeutics SAS, Elancourt 78990, France; ^3^INSERM UMR935, University of Medicine Paris Sud 11, Orsay, France; ^4^Vet Tox Path Consulting, Theizé 69620, France; ^5^Institut de Recherche Biomédical des Armées, Unité de Thérapie Cellulaire et Réparation Tissulaire, Brétigny sur Orge 91223, France

## Abstract

**Objective:**

To assess the clinical value and safety of the application of allogeneic equine oral mucosa mesenchymal stromal cells (OM-MSCs) to wounds. *Animals*. 8 healthy adult horses without front limb skin lesions or musculoskeletal disease. *Procedures*. Stem cells were isolated from the oral mucosa of a donor horse. Horses were subjected to the creation of eight full-thickness cutaneous wounds, two on each distal forelimb (FL) and two on both sides of the thorax (TH). Each wound was subjected to one out of four treatments: no medication (T1), hyaluronic acid- (HA-) gel containing OM-MSC (T2), HA-gel containing OM-MSC secretome (T3), and HA-gel alone (T4). Gross macroscopic evaluation and laser digital photographic documentation were regularly performed to allow wound assessment including wound surface area. Full-thickness skin punch biopsy was performed at each site before wound induction (D0, normal skin) and after complete wound healing (D62, repaired skin).

**Results:**

All wounds healed without adverse effect at D62. Distal limb wounds are slower to heal than body wounds. OM-MSC and its secretome have a positive impact on TH wound contraction. OM-MSC has a positive impact on the contraction and epithelialization of FL wounds. No significant difference between wound sites before and after treatment was noted at histological examination. *Conclusion and Clinical Relevance*. Using horse cells harvested from oral mucosa is a feasible technique to produce OM-MSC or its secretome. The gel produced by the combination of these biologic components with HA shows a positive impact when applied during the early stage of wound healing.

## 1. Introduction

Wound healing is a dynamic process that proceeds through a carefully orchestrated interaction of cellular and molecular events, which start whenever there is a break in tissue integrity. However, second, intention wound repair in horse limbs often progresses toward complications, including chronic nonhealing wounds and development of exuberant granulation tissue (EGT) [1]. Impaired wound healing occurs mostly on the distal part of the limbs, often becoming chronic wounds with a lack of epithelial cover [2]. Current knowledge indicates that nonhealing wounds or development of EGT have many contributing factors, including reduced angiogenesis [3, 4], persistent secretion of growth factors leading to a fibroproliferative response [5], and an imbalance in collagen homeostasis [6].

In horses, such fibroproliferative disorders [7] are responsible for poor healing in the distal limbs, limiting an athletic career and at the origin of expensive treatments.

There is accumulating evidence that stem cell therapy can facilitate wound healing [8–10]. Mesenchymal stromal cells (MSCs) exhibit immunomodulatory, anti-inflammatory, reparative, and regenerative properties, suggesting that they may be used in various inflamed or injured tissues. It has been demonstrated that MSCs may decrease toxic inflammation and reduce tissue injury after cardiac, kidney, and liver disease in animal models [11–13] and that they have a beneficial effect on wound repair in rodent models [14–18] and horses [19, 20]. These cells can be isolated from various tissues and modulate wound healing through the release of several paracrine factors, enzymes, and immunomodulatory cytokines [21]. In addition to these properties, they have shown the ability to release into the extracellular environment a number of vesicles containing multiple factors with therapeutic efficacy largely demonstrated in different animal models [22, 23]. These lipids, nucleic acid, and proteins (growth factors, chemokines, cytokines, adhesion molecules, and proteases) secreted by the cell into the extracellular space are called the secretome.

In this study, we evaluated the application of equine allogeneic OM-MSC or OM-MSC secretome to experimentally induced thoracic and distal limb wounds in horses. We hypothesized that in horses these treatments are safe and have a positive impact on wound healing when compared with HA, vehicle treatment, and untreated controls.

## 2. Materials and Methods

### 2.1. Animals

The study was approved and performed according to the guidelines of the French Animal Ethics Committee (APAFIS#5264-2016042716164161). Eight healthy mixed-breed adult horses (four geldings and four mares) of varying ages (range: 9–15 years), free of front limb lameness and of any scars or skin disease, were included in the study. On the day of surgery (D0) and for the next sixteen days (D1-D16), horses were housed in box stalls and then in outdoor small paddocks until the end of the study (D17-D90). Horses were fed with 10 kg of hay twice daily and had free access to water.

### 2.2. Study Design

This unicentric study was a double-blind, randomized trial. After a one-week acclimation period, all horses underwent standing surgery (D0) to induce four skin wounds at two different body sites (TH and FL). A four days' treatment period (D1 to D4) was followed by an evaluation period until D90. Treatments were applied to the wounds under blind conditions, each horse being its own control.

### 2.3. Isolation and Culture of OM-MSC

Oral mucosa-derived mesenchymal stromal cells were isolated from a two square centimeter tissue biopsy obtained from the oral mucosa of a donor horse. Small cut pieces of the biopsies were digested by a solution containing 15 mL dispase II 240UI (Roche), 300 *μ*L clinical grade MTF collagenase II (Invitrogen) complemented with amphotericin (2.5 *μ*g/ml), penicillin (100 IU/ml), and gentamicin (50 *μ*g/ml). The sample was digested at 37°C for 2 h30. After digestion, the suspension was sieved and the filtrate was centrifuged at room temperature at 400 g for 10 min. The resulting cell pellet was taken up in an alpha MEM culture medium (Gibco) at 2000 cells/cm^2^. This medium was changed after 48 h with a low concentration of amphotericin (1 *μ*g/ml) and then changed every 48 h until 80% confluence. MSCs obtained from the first passage were used for in vivo experiments. Trilineage differentiation of OM-MSC was verified according to the R&D Systems kit recommendations (R&D Systems).

#### 2.3.1. Flow Cytometry of Equine OM-MSC

**Figure 1:**
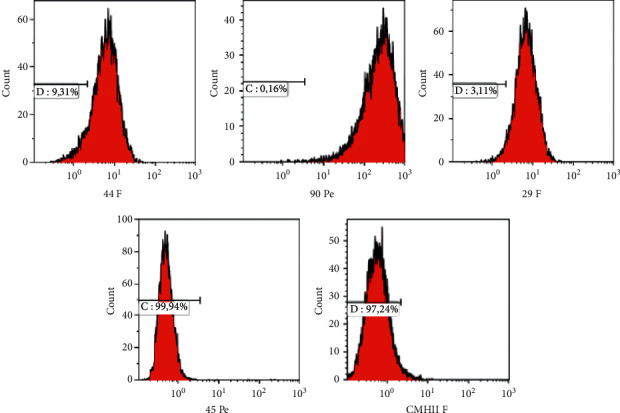
Phenotypic characterization of OM-MSCs. These results show that the cells express a typical MSC phenotypic profile: CD45-/CD44+/CD90+/CD29+/MHC II.

Aliquots of 200,000 cells per well of a 96-well plate were prepared in cytometry buffer. Cells were then incubated for 20 min at 4°C in the dark with different flow cytometry antibodies before being washed with PBS twice at 400 g for 2 min. Cells were suspended in PBS for further flow cytometry analysis (Figure 1). The antibodies used were specific for equine MSCs markers: CD44, CD90, CD29, CD45, and MHC II. The analysis was performed using a Beckman flow cytometer and data treated. (Kaluza Beckman software).

#### 2.3.2. Preparation of HA-Gel

Cells were harvested with Trypsin, washed with PBS, and resuspended in HA-gel to be administered within 24h. HA (HTL biotechnology, Mw = 240 kDa) was dissolved in water (2% final concentration) and stirred at 250 rpm, 60°C, for 12 hours, and then mixed with MSCs. A total of 500.10^3^ cells were obtained and used for HA-gel preparation at a concentration of 50.10^3^ cells/cm^3^ of gel.

### 2.4. Wound Creation

**Figure 2:**
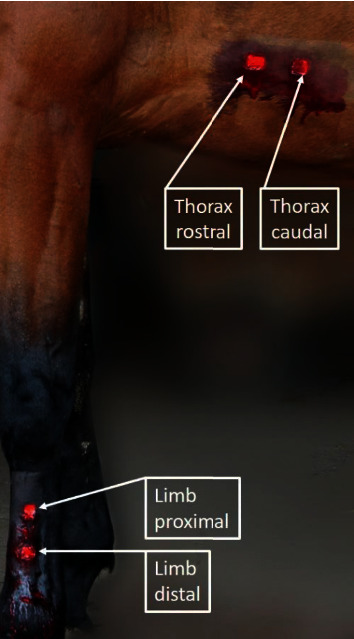
Experimental design. Two wounds were created on both FL and the TH 30 cm behind the tip of the elbow on a balanced horse. Treatment application was randomized in terms of right or left side, cranial-caudal (TH), or proximal-distal (FL) wound assignment. The four treatments (OM-MSC or its secretome embedded in an HA-gel, HA-gel alone, or no treatment) were applied daily two (G1) or four (G2) times, starting 24 hours after wound creation.

On D0, horses were sedated with detomidine (0.01 mg/kg IV) and butorphanol tartrate (0.04 mg/kg IV). Local anesthesia was obtained with a proximal lateral metacarpal half-ring block on both forelimbs and a line block on the thorax using a 2% solution of lidocaine hydrochloride. Surgical sites were clipped and aseptically prepared. A total of eight 2.5 × 2.5 cm full-thickness skin wounds were created: two on the dorsolateral aspect of each metacarpus III of both FL and two on the TH spaced by 4 cm in an area located 30 cm behind the tip of the elbow on a balanced horse (Figure 2). A template was used to standardize the creation of wound areas. Wounds were not covered after surgery to mimic spontaneous trauma in the field.

### 2.5. Wound Treatment

All wounds of the thorax and front limbs were submitted to the application of one out of four treatment options (T1 to T4). For each of the two body regions, thorax or front limb, one wound was untreated (T1) to serve as a control, one wound was treated with OM-MSC embedded in HA-gel (T2), one wound was treated with OM-MSC secretome embedded in HA-gel (T3) (StemCream®, StemT, France), and one wound was treated with HA-gel (T4) alone. Each individual clinical record includes a wound map with different colors (white, red, green, and blue) attributed to each treatment blinded to the clinicians in charge of the study. Treatments were shipped in a temperature-controlled cooler directly from the manufacturer to the Lyon Equine Research Centre in sterile syringes labeled with one of the four colors. In this manner, the model was guaranteed a double-blind trial. Horses were randomly divided into two groups (G1 and G2). The first group received all four treatments twice with 48-hours intervals (D1 and D3) and G2 received the treatment four times during four consecutive days (D1, D2, D3, and D4).

### 2.6. Wound Dressing

On D1, all thoracic wounds were dressed with a nonadherent permeable dressing secured with a cohesive bandage. Dressings were changed at D2, D3, and D4 and removed on D6, 48 h after the last treatment (D5 for G1 and D6 for G2).

On D1 after treatment application, all limb wounds were dressed with a nonadherent permeable dressing secured with sterile conforming cotton gauze and held in place with a cohesive bandage. Dressings were changed at D2, D3, D4, and every 4 days until D16. Horses were then placed in a small outdoor paddock, without any bandages.

### 2.7. Clinical Evaluation

To detect any discomfort, a daily individual general clinical examination was performed during the box rest period (16 days) and the paddock period (74 days). The final clinical examination was performed at D90. Horses did not receive any other medication during the study.

### 2.8. Wound Noninvasive Assessment

Before the first treatment application 24 h after surgery (D1), and on D3, D5, D7, D11, D15, D19, D23, D28, and once a week until D90, gross visual evaluation and digital photography were achieved for all wounds.

Gross visual wound evaluation consists in assessing wound inflammation, granulation tissue, epithelialization, contraction, and detecting any abnormal evolution such as infection. Scoring from 0 to 4 was created and used to describe the wound at each evaluation period:

  Score 0: original wound size 2.5 × 2.5 cm
  Score 1: increased wound size with all four wound edges visible
  Score 2: increased wound size with at least one wound edge covered with granulation tissue
  Score 3: reduction in wound size without epithelialization
  Score 4: reduction in wound size with a minimum of one visible border of epithelialization

**Figure 3:**
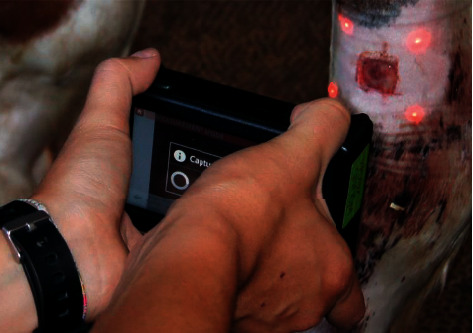
Digital image. The four laser beams of the device were projected and aligned outside of the wound border. After connecting the camera to a computer, images were treated by specific software.

**Figure 4:**
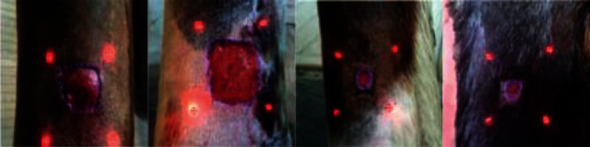
Digital image. The laser beams projected by the camera on the wound surface (red) draw a square shape allowing the clinician to delineate a precise wound perimeter (blue) and the software to calculate a surface area. This figure shows four types of wounds at different stages of the healing process.

Digital image, using a Laser Digital Wound documentation device (Wound Zoom Inc, WI 54481, USA) of each wound, was obtained to get precise wound area measurements. This device was provided with four laser beams projected and aligned slightly outside of the wound border (Figure 3). The correct alignment of the laser beam allowed tracing the wound perimeter (Figure 4). After connecting the camera to a computer, the images were treated by specific software, allowing precise calculation of wound circumference and surface area. Before each digital documentation, the wound periphery was gently cleaned with a sterile saline solution to better visualize the wound edges.

### 2.9. Wound Invasive Assessment-Histology

For histologic evaluation, a full-thickness biopsy sample was obtained during wound creation (normal skin) and two months later (D62, repaired wound). An 8 mm diameter biopsy punch was used to obtain these samples. For each wound, one central and one wound edge biopsies were collected. Samples were fixed in neutral buffered 10% formalin. After preparation for histologic examination, sections were scored 0–4 for each of the four categories: degree of epithelialization, granulation tissue, inflammatory cell infiltration, and neovascularization. For all scores, 0 was assigned to sections lacking the histologic feature, and 4 was attributed when the feature was significant.

### 2.10. Statistical Analysis

Statistical analysis was performed with statistical software (R software environment version 3.4.1 “Single Candle”). Wound scores were statistically treated individually by type of wound: FL and TH. The experiment map of the wound evaluation was performed by two-way ANOVA with decomposition on time factor (9 time points from D0 to D28) and on the treatment factors with four conditions of treatment: control (T1 to T4). The absence of interaction between the evaluated factors was verified during this analysis. To estimate the effect of each treatment, wound scores were analyzed by adjustment of a linear model. Finally, frequency tables of scores were generated by Pearson's Chi-square test. Scatterplots and bar plots were performed with ggplot2 graphical definition [24]. For all biological hypotheses, the rejection of the null hypothesis during statistical tests was taken into account for an error alpha less than 0.05.

## 3. Results

### 3.1. Flow Cytometry of Equine OM-MSC

The presence of pure MSC in the culture was demonstrated by phenotypic characterization, osteogenic and chondrogenic differentiation, and CFU-F formation.

### 3.2. Clinical Evaluation

None of the horses have shown discomfort or lameness after wound creation. All wounds healed completely by the end of the study (D90) without adverse effects.

### 3.3. Wound Noninvasive Assessment

Gross wound-healing variations were observed within the first month after wound creation, and no significant differences were collected after this period. On the third day after wound creation (D3), based on digital photography results, the overall circumference of most wounds (53/64) was smaller compared to its original size on D0. Between D5 and D7, an increase in size was noticed in 43 wounds (43/64).

For TH, a maximum decrease in wound surface area was then observed at D11 (30/32), and by D23, all TH had reached full healing or a wound surface area less than 0.4 mm^2^.

For FL wounds, a progressive increase in circumference and surface area was still observed at D11 and until D19 (26/32). After D19, a decrease was observed in all FL wounds, and full healing was recorded at D60.

#### 3.3.1. Wound Contraction

**Figure 5:**
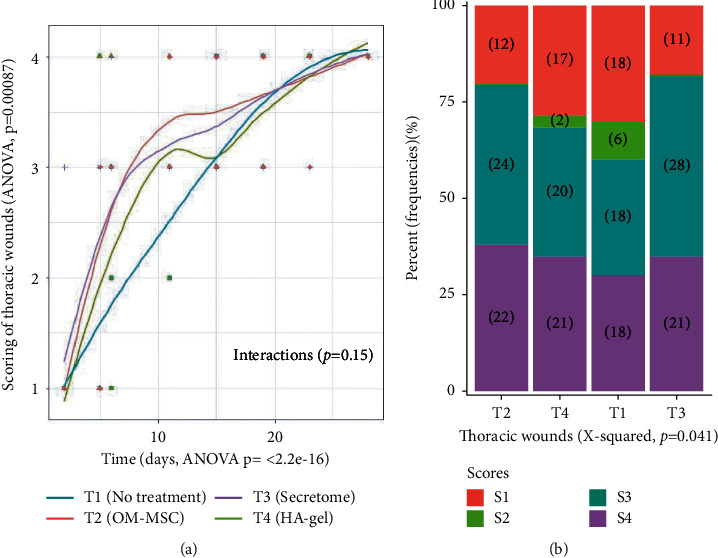
Thoracic wound noninvasive assessment. (a) Gross wound evaluation scoring evaluated by a Fisher two-way ANOVA. It shows a significant increase in score with time. (b) Cross frequencies between the levels of the wound and the different treatments investigated with bar plot and Pearson's Chi-square test. T2 and T3 treatments applied to TH wounds showed an increase in level 3 score corresponding to increased wound contraction.

Eleven days after wound creation (D11), no epithelialization border was noticed in any TH wounds, but their overall surface area was significantly smaller in horses of G1 treated twice with OM-MSC gel (T2) or with OM-MSC secretome gel (T3) compared with T1- and T4-treated wounds. This observation was not recorded in G2 treated four times during four consecutive days. For TH wounds, a two-way ANOVA could not detect any significant difference in surface area evolution, between treatments. The gross wound evaluation score was evaluated by a Fisher two-way ANOVA (factor time with nine time points from day 0 to day 28 and factor treatment with four conditions T1, T2, T3, and T4). This analysis shows a significant increase in scores with time (Figure 5(a), ANOVA *p* < 2.2.10 − 16), suggesting that these scores are well adapted to assess thoracic wound progression with time during the first 28 days of wound healing. A significant effect of treatment on TH wounds is present (Figure 5(a), ANOVA, *p*=0.00087). Linear model analysis revealed no significant difference between control (T1) and HA-gel (T4) (*p*=0.1824) but identified a statistically significant effect of T2 (*p*=0.000748) and T3 (*p*=0.000889) compared to T1. T2 and T3 scores were higher than the control score (T1) during the first 28 days of wound healing. Cross frequencies between the levels of TH wound and the different treatments were investigated with bar plot and Pearson's Chi-squared test, which confirmed the significant effect of the treatments on the scores (Figure 5(b), *X*-squared test, *p*=0.041). T2 and T3 treatments applied to TH wounds showed an increase in level 3 score corresponding to increased wound contraction.

#### 3.3.2. Wound Epithelialization

**Figure 6:**
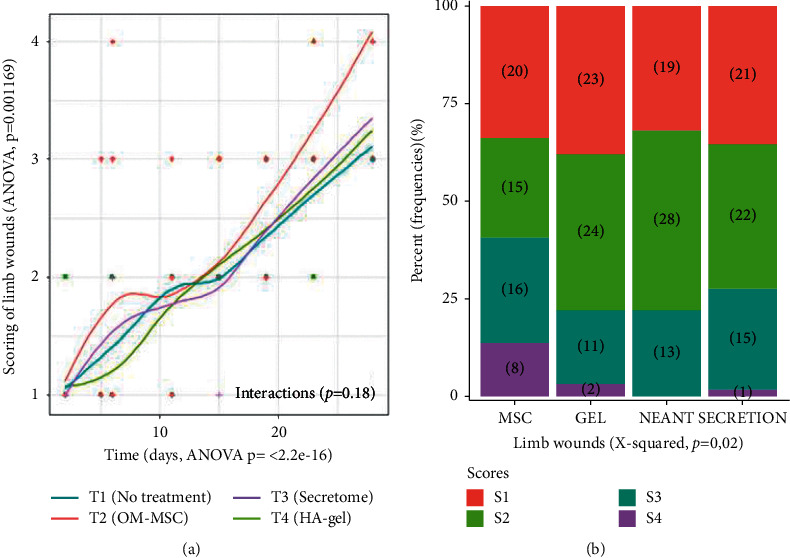
Limb wound noninvasive assessment. (a) A Fisher two-way ANOVA revealed a significant effect of treatment on FL wounds (*p*=0.0012). Linear model analysis reveals a significant difference between T2 (OM-MSC) and T1 (control) (*p*=0.001289). (b) OM-MSC shows an increase in the frequency of scores 3 and 4, respectively, related to an increase of contraction and contract on epithelialization of the FL wound.

All FL wounds initially enlarged in size before reducing their surface area, starting at D23. A Fisher two-way ANOVA (factor time with nine time points from D0 to D28, and factor treatment with four conditions T1 to T4) assessed the scoring for both FL and TH wounds. This score analysis revealed a significant effect of treatment on FL wounds (Figure 6(a), ANOVA, *p*=0.0012). Linear model analysis revealed no significant difference between T1 and T4 (*p*=0.725), nor between T1 and T3 (*p*=0.604). However, a significant difference between T2 (OM-MSC) and T1 (control) was observed (*p*=0.001289). This T2 effect on FL wounds is more pronounced before D10 and regressed from D15 until the end of the study. To understand which score is involved in this OM-MSC effect, a frequency table was generated in a bar plot by Pearson's Chi-squared test which confirmed the significant effect of treatment on the scores (Figure 6(b), *X*-squared *p*=0.02). T2 on FL wounds showed an increase in the frequency of scores 3 and 4. These scores are, respectively, related to an increase of contraction and contraction epithelialization of the wound.

### 3.4. Wound Invasive Assessment-Histology

When compared with controls (T1), the mean histological score obtained for TH and FL wounds was higher at sites receiving HA-gel treatment alone (T4). Sites that received T3 (OM-MSC secretome) had a lower mean score and the ones receiving T2 (OM-MSC) had the lowest mean score. Mean scores were higher at TH sites compared to FL sites and the cranial TH site had a marginally higher score compared to the caudal TH site.

## 4. Discussion

Comparison between wound sites shows that the process of reduction in wound surface area takes longer in FL compared to the TH. After 23 days, all TH wounds had nearly achieved their healing process, whereas FL wounds only started their decrease in surface area size. This faster healing process on the TH compared to the wound on an FL (23 days versus 60 days) is in agreement with previous results showing a slower contraction rate on the distal compared to the proximal part of the limb (shoulder) [25, 26]. Macroscopic wound scoring shows a higher degree of wound contraction (score 3) at the TH site, explaining why scars are usually smaller on the body compared to the limbs of a horse.

Given the difficulty to treat equine limb wounds, the use of regenerative therapies has been suggested as a new promising treatment. Currently, the therapeutic use of MSCs in equine practice is principally dedicated to treating musculoskeletal disorders including tendon, ligament, and joint disorders [27–29]. This study assesses the clinical value and safety of the application of allogeneic OM-MSC or its secretome in wound healing of horses.

Eleven days after wound creation, a significant decrease in the circumference and surface area was described only for TH wounds treated with HA-gel containing OM-MSC or its secretome. We can conclude from these observations that a beneficial effect can be obtained with these regenerative medicine treatments when used at an early stage of the wound healing process. These results also suggest the existence of a probable therapeutic window for drug application. We assume that, in the first phase of healing, the presence of OM-MSC or its secretome leads to amplification of the wound healing process. This hypothesis is in agreement with the assumption of Textor [20] observing a dramatic increase in the expression of COX-2 when MSCs are injected in equine wounds one week after creation. These results are also in concordance with previous data reporting that priming of MSCs by inflammatory signals is required to have a therapeutic effect [28].

Transdifferentiation and paracrine effects could explain the action mechanisms of MSCs in wound healing. These cells can act as building blocks via their characteristic developmental plasticity, which enables differentiation from one cell type to another, also known as transdifferentiation [14]. The capacity for differentiation along osteogenic, adipogenic, chondrogenic, and myocytic lineages has been largely demonstrated [30, 31]. Recent reports in humans, mice, and horses have also shown that induced pluripotent stem cells can successfully be differentiated into the keratinocyte lineage [32] and into endothelial cells [33]. Despite having the capabilities of engraftment and differentiation into various cell types, the major tool to modulate the wound healing process seems to be the secretome [23]. This paracrine-mediated effect is possible because MSCs display a rich secretory profile, which is enhanced by exposure to inflammatory signals. It contains factors capable of modulating the activation, migration, and proliferation of several cells involved in the healing process after a break in tissue integrity [29, 34]. Through these paracrine interactions, MSCs can regulate collagen production, enhance skin regeneration, promote angiogenesis, and increase wound closure rates [35, 36]. In this study, the positive effect of therapy described on TH wounds could not detect any clinical differences between OM-MSC and its secretome. *In vitro,* it has been demonstrated that the secretome enhances the healing process through activation of the inflammatory cascade and by stimulation of fibroblasts, keratinocytes, and vascular epithelial cell proliferation [37]. To our knowledge, in horses, there are no studies assessing the secretome in the repair process of wounds, nor comparative studies between MSCs and their paracrine factors.

Histological scoring in this study revealed that the mean histological scores obtained for TH wounds receiving HA-gel with OM-MSC and HA-gel alone were about the same. This shows that HA-gel is a good candidate for being the carrier of OM-MSCs or its secretome. Histology tells us the state of the skin at a specific moment. In this study, it represents the end of the healing process. This explains why we cannot highlight the encouraging result of applying OM-MSC or its secretome at the beginning of the healing process.

Despite the beneficial effect on wound healing of OM-MSC applied twice in G1, this effect was not observed when treatment was repeated four times in G2. It suggests a potential cumulative dose effect of treatment or interference with bandage changes in the early phase of wound healing (4 bandage changes in four days instead of two). We postulate that cumulative treatment can contribute to delayed healing. Further research into inflammatory protein expression by OM-MSCs or their paracrine targets during wound repair needs to be performed to clarify the mechanism of action.

Bandages were definitely removed from all FL at D16, but all wounds continued to show a progressive increase in the surface area until D19 before decreasing in size for 90% of the wounds by D23. Bandaging could be an element to the detriment of a normal wound healing process. In experimentally induced wound healing studies, bandages are intentionally used to create slow healing wounds and EGT on the distal limbs of horses [38]. Bandaging lowers surface oxygen tension and creates hypoxia, which then drives further angiogenesis and fibroplasia and creates EGT. In another study [20], wound limbs were unbandaged to avoid interference with the healing process.

Oral mucosa mesenchymal stromal cells and OM-MSC secretome used in this study were applied locally in an HA-gel to reduce the low efficacy of homing and migration of MSc to the target lesion reported during systemic delivery [39]. Histologic findings revealed that at D62 FL wounds treated with HA-gel are the most rapid ones to heal. These results show a potentially positive effect on wound healing of HA alone. Hyaluronic acid is a natural polymer largely used in designing biomaterials for stem cell delivery. It shows multiple properties including enhanced angiogenesis, maintaining cell survival, and reepithelialization in a murine model [21, 40]. Contraction and epithelialization following HA-gel application on cutaneous wounds have been previously demonstrated [41–43], which is why this medium was chosen for the administration of OM-MSC and its secretome. This positive effect of the transport medium was not observed in another study using a fibrin-gel medium [20].

All TH wounds healed without EGT or excessive scar formation. In contrast, some distal limb wounds developed mild EGT, independently of the treatment applied. This is a well-described phenomenon in equids [1]. Continuous bandaging for sixteen days' postwound creation was probably a major contributing factor to the development of EGT. A TH bandage was only applied on top of the wounds for six days starting with the first treatment application. Finding a specific biomarker for wound healing status would probably help treat an impaired process in horses such as the development of EGT. Several cellular events and mediators associated with wound healing can serve as biomarkers such as IL-1, IL-6, and MMPs [44]. However, currently, no valid factors have been developed in equids; therefore, they have not been used in this study.

Cells can be readily derived from a variety of autologous or allogeneic tissues. In horses, umbilical cord and placenta tissue origin have shown some efficacy in various applications, comparable to traditional sources such as MSCs isolated from bone marrow or adipose tissue [20, 45–47]. In this study, allogeneic OM-MSC or its secretome was used for the first time in horses. In human medicine, research studies on the oral mucosa and gingiva [48] are performed given that oral wounds heal faster and with better scar quality compared to skin wounds [49, 50]. Oral wounds are usually scarless, similar to fetal wound healing. In human patients, the difference in wound healing and final scar quality might be related to differences in MSC and their ability to respond to intrinsic (autocrine) and extrinsic signals, such as human salivary histatin [51, 52], epidermal growth factor, and transforming growth factor *β*1 that is mostly implicated in the deposition of extracellular matrix [53]. Several studies of MSC, derived from human oral mucosa and gingiva, applied in human patients and animals, have shown better wound healing [54] with an immunomodulatory and anti-inflammatory effect [55]. Research in this field needs to be related to a better understanding of normal acute wound repair, a highly dynamic cascade of cellular signaling, and behavioral events involving multiple inflammatory mediators [56, 57]. Any perturbation to this system leads to aberrations such as excessive scarring or failure to heal.

## 5. Conclusions

In equine medicine, observation of the highly regenerative capacity of horse oral mucosa suggests the existence of a robust stem cell population in that tissue [48]. This assumption is confirmed in this study where OM-MSC and its secretome showed a positive impact on wound healing. More work needs to be directed in understanding the best therapeutic window and the best bandage protocol for optimization of these innovative treatments. However, these results already suggest that, during the early stage of healing, an HA-gel containing OM-MSC or its secretome induces a more rapid contraction profile in TH wounds and that OM-MSC gel has a stimulating effect on FL wound contraction and epithelialization process.

## Data Availability

The datasets used and/or analyzed during the current study are available from the corresponding author on reasonable request.
